# Modulation of axon excitable domains by astrocytes and microglia

**DOI:** 10.3389/fncel.2025.1749755

**Published:** 2026-01-08

**Authors:** Juan José Garrido

**Affiliations:** 1 Instituto Cajal, Centro de Neurociencias Cajal, CSIC, Madrid, Spain; 2 Centro de Investigación Biomédica en Red sobre Enfermedades Neurodegenerativas (CIBER-ISCIII), Madrid, Spain

**Keywords:** astrocytes, axon, axon initial segment, microglia, nodes of Ranvier

## Abstract

Neuronal communication depends on neuronal polarity and the integrity of axonal excitable domains, including the axon initial segment (AIS), nodes of Ranvier, and presynaptic terminals. In addition to the influence of neuronal input on their function and plasticity, recent evidence suggests that glial cells play a significant role in regulating these domains under both physiological and pathological conditions. In this context, this review focusses on the roles of astrocytes and microglia in the physiological modulation of the AIS and nodes of Ranvier and how these glial cells are involved in several pathological contexts, beyond its participation in the tripartite synapse. The AIS and nodes of Ranvier are not only essential for the initiation and propagation of neuronal signals but also serve as key sites of interaction with astrocytes and microglia. These interactions are crucial for maintaining neuronal excitability and overall neural circuit health. Disruptions in the interactions between glial cells and the AIS or nodes of Ranvier—whether caused by injury or disease—can profoundly affect central nervous system (CNS) function, emphasizing the importance of this dynamic relationship in both normal and pathological contexts. Recent studies have highlighted the roles of astrocytes and microglia in contacting the AIS and nodes of Ranvier, contributing to their structural plasticity, as well as in maintaining their homeostasis through the secretion of signaling factors and the regulation of ion concentrations in their microenvironment. However, the mechanisms underlying these regulatory processes remain largely unknown, and further research is required to elucidate how these interactions influence axonal physiology and contribute to axonal pathology.

## Introduction

The nervous system is a highly complex and dynamic network that governs bodily functions, cognition, and behavior by transmitting electrical and chemical signals. Central to this intricate communication system are neurons and glial cells, which orchestrate the regulation and flow of information. Within each neuron lies a specialized region known as the Axon Initial Segment (AIS), a structurally and functionally distinct domain that plays a pivotal role in action potential initiation and neuronal polarity. Together with the AIS, the structurally similar nodes of Ranvier contribute to the saltatory conduction along myelinated axons, increasing conduction velocity and minimizing energy expenditure. Surrounding and supporting these neurons are glial cells, a diverse group of non-neuronal cells essential for maintaining homeostasis, forming myelin, and providing structural and metabolic support and protection for neurons.

The AIS is located at the junction between the neuronal soma and the axon and is characterized by a dense cytoskeletal and molecular architecture (Rasband, 2010). Structurally, the AIS acts as a gatekeeper controlling the entry of proteins toward the axon through: (1) a membrane diffusion barrier mechanism (Nakada et al., 2003), and (2) the control of anterograde and retrograde traffic to permit axonal cargoes while excluding those containing somatodendritic proteins (Kuijpers et al., 2016). These processes confer upon the neuron its characteristic somatodendritic-axonal polarity. Functionally, the AIS is the place of action potential generation, supported by a high density of voltage-gated ion channels (Kole et al., 2008). Given the importance of these functions, the loss of AIS integrity compromises axonal identity and neuronal viability (Schafer et al., 2009). Although the AIS is a highly stable structure, it exhibits a remarkable degree of plasticity, including length changes (shortening or elongation), positional shifts (proximal or distal movement along the axon), and molecular remodeling (modification of ion channel composition or anchoring proteins). These adaptations serve as homeostatic responses to chronic changes in neuronal activity and contribute to generating adapted electrophysiological output signals in response to various intrinsic and extrinsic physiological or pathological inputs (Grubb and Burrone, 2010; Kuba et al., 2010; Del Puerto et al., 2015). Despite the clear relevance of AIS integrity and plasticity to neuronal adaptation and brain diseases, the intrinsic and extrinsic mechanisms underlying AIS plasticity remain mostly unknown. Several structural and functional changes have been identified in response to altered input activity or its absence, as well as in association with glial cell mechanisms.

The nodes of Ranvier are essential for maintaining and propagating the action potentials generated at the AIS in myelinated axons. These axonal domains lack myelin and are flanked by regions where oligodendrocytes (in the CNS) or Schwann cells (in the PNS) contact the axon, forming the paranode and juxtaparanode (Eshed-Eisenbach et al., 2023), while the AIS is in contact with the myelin sheath in its distal region (para-AIS). Like the AIS, nodes of Ranvier and their surrounding myelinated structures exhibit adaptive capacity through modifications in the interactions between oligodendrocytes/Schwann cells and axons, leading to changes in nodal properties (Suminaite et al., 2019). In this context, perinodal astrocytes in the CNS and microglia play an important role, which is still not well understood.

## Axon initial segment and nodes of Ranvier structure and function

The AIS possesses specific membrane, cytoskeleton, and voltage-gated ion channel characteristics compared to the axon and the somatodendritic compartment. Several recent reviews have thoroughly described AIS structure and function (Rasband, 2010; Bender and Trussell, 2012; Leterrier and Dargent, 2014). Briefly, the AIS contains a highly enriched concentration of voltage-gated sodium (Na_v_) and potassium (K_v_7.2/3, K2P or K_v_1.1) channels, anchored by specific scaffold proteins such as ankyrin-G or PSD-93 (Garrido et al., 2003; Pan et al., 2006; Ogawa et al., 2008; Luque-Fernandez et al., 2024), through specific protein motifs in ion channels (Garrido et al., 2003; Pan et al., 2006; Luque-Fernandez et al., 2024). Voltage-gated ion channels expression is dependent on neuronal type, axonal myelination and even animal species (Debanne et al., 2011; Kuba et al., 2015). AnkyrinG is the main protein in this scaffold, and its loss leads to AIS disruption and absence of neuronal polarity (Schafer et al., 2009). It anchors membrane proteins, such as neurofascin 186 (Zonta et al., 2011), sodium or potassium voltage-gated ion channels. AnkyrinG binds to microtubules through end-binding 1/3 (EB1/3) proteins (Leterrier et al., 2011); meanwhile, ankyrinG interacts with βIV-spectrin and anchors the scaffold to the actin cytoskeleton. Axon initial segment microtubules are characterized by higher tubulin acetylation and detyrosination, which allows the binding of kinesin-1 and axonal cargo entry to the axon (Konishi and Setou, 2009). In addition, TRIM46 is in charge of AIS microtubule fasciculation (Harterink et al., 2019), and other microtubule-associated proteins (MAPs) are present at the AIS, such as tau or MAP7D2 (Pan et al., 2019). The AIS actin cytoskeleton contains actin rings, sparse actin fibers, and actin patches that participate in AIS plasticity (Micinski and Hotulainen, 2024).

The structure, formation, and function of the nodes of Ranvier have been thoroughly described previously (e.g., Poliak and Peles, 2003). The nodes of Ranvier are enriched in voltage-gated sodium channels, scaffold proteins such as ankyrinG and βIV-spectrin, and adhesion molecules such as Neurofascin 186. The formation of the axoglial junction in the paranodal region requires Caspr and contactin proteins, which link to the cytoskeleton through protein 4.1B. The connection between the paranode and the myelinating glial cells is maintained by contactin in the paranode, which binds neurofascin 155 in glial cells.

## Axon initial segment and nodes of Ranvier regulation by neuronal intrinsic mechanisms

AIS plasticity serves as a mechanism that allows neurons to modulate their initial signal output, adapting it to their overall synaptic input. Although our knowledge of the regulatory mechanisms governing the AIS remains limited, several studies have demonstrated the involvement of specific neuronal molecular mechanisms in maintaining AIS integrity, function, and plasticity. These include kinases such as GSK3, CK2, or cdk5, phosphatases such as calcineurin, and proteases, such as calpain (Brechet et al., 2008; Schafer et al., 2009; Evans et al., 2013; Tapia et al., 2013; Del Puerto et al., 2015; Jahan et al., 2023). During the early stages of AIS development, endocytic mechanisms play a role in its assembly (Torii et al., 2020). Changes in AIS plasticity and molecular composition are associated with the regulation of presynaptic neurotransmitter receptors and variations in dendritic morphology. For example, decreased expression of the cannabinoid receptor CB1 leads to shorter dendrites during early neuronal development and a reduction in ankyrinG concentration at the AIS (Tapia et al., 2017). Conversely, in pyramidal neurons, apical dendrite diameter inversely correlates with the distance of the AIS from the soma (Hamada et al., 2016). In this context, deprivation of presynaptic activity results in AIS elongation and increased neuronal excitability, affecting Nav channels distribution (Kuba et al., 2010), while changes in electrical activity can alter the location of the AIS (Grubb and Burrone, 2010). Certain neurotransmitters, including GABA, dopamine, or serotonin, also regulate action potential initiation. Dopamine receptor activation downregulates Ca_v_3 channel activation, thereby reducing neuronal output (Bender et al., 2010). Serotonergic regulation of 5-HT1A receptors can modulate AIS HCN channels, modifying action potential threshold (Ko et al., 2016). GABA at the AIS is likely inhibitory for action potential initiation (Lipkin and Bender, 2023), and ATP can modify AIS composition through calcium-dependent mechanisms (Del Puerto et al., 2015). These intrinsic neuronal mechanisms that modulate AIS physiology, integrity, and plasticity also depend on extrinsic regulatory processes by glial cells.

Much less is known about the intrinsic axon- and myelin-dependent regulation of nodes of Ranvier. Some studies have described variations in the geometry of nodes of Ranvier along the axons of globular bushy cells (GBCs) (Ford et al., 2015). Evidence also points to a regulation of ion channel expression mediated by neuronal activity (Grundemann and Clark, 2015). Other studies propose an inside-out model, in which the axonal cytoskeleton contributes to myelin remodeling, enabling myelin sheath elongation or retraction and dynamic regulation of node size (Suminaite et al., 2019). Conversely, there is also evidence supporting the role of oligodendrocytes in regulating axonal properties, thereby influencing the size and function of the nodes of Ranvier.

## Glial cell regulation of the axon initial segment and nodes of Ranvier

Glial cells are now recognized as active participants in virtually every aspect of nervous system function. They are broadly classified into several major types (astrocytes, oligodendrocytes, microglia, and Schwann cells), each fulfilling distinct and essential roles. Astrocytes regulate the extracellular ionic and neurotransmitter environment, modulate synaptic transmission, and contribute to the maintenance of the blood–brain barrier (Araque et al., 2001). Oligodendrocytes and Schwann cells are responsible for myelin formation in the central and peripheral nervous systems, respectively, thereby enabling rapid action potential conduction (Nave and Werner, 2014). Microglia act as the resident immune cells of the brain, responding dynamically to injury and infection (Wolf et al., 2017). The interaction between the AIS and glial cells is increasingly recognized as a key factor in neural development, plasticity, and pathology. Glial cells modulate the structure and function of the AIS through multiple mechanisms, including ion buffering, neurotransmitter uptake, inflammatory signaling, and direct structural interactions. For example, astrocytes influence AIS stability and function by regulating ion homeostasis and providing metabolic support (Sanz-Galvez et al., 2024), whereas microglial activity can induce AIS remodeling in response to inflammation or injury-related signals (Clark et al., 2016). The nature of this regulation is context-dependent, varying with developmental stage, neural activity, and pathological conditions. Disruptions in AIS structure and glial function are implicated in a variety of neurological disorders, including epilepsy, multiple sclerosis, and neurodegenerative diseases (Garrido, 2023). These findings underscore the critical importance of glia-AIS interactions in both health and disease. Advancing our understanding of their individual roles and complex bidirectional communication offers deep insight into the mechanisms underlying neural communication and may reveal new targets for therapeutic intervention in neurological diseases.

## Astrocyte-mediated regulation of the AIS and nodes of Ranvier

**Figure 1:**
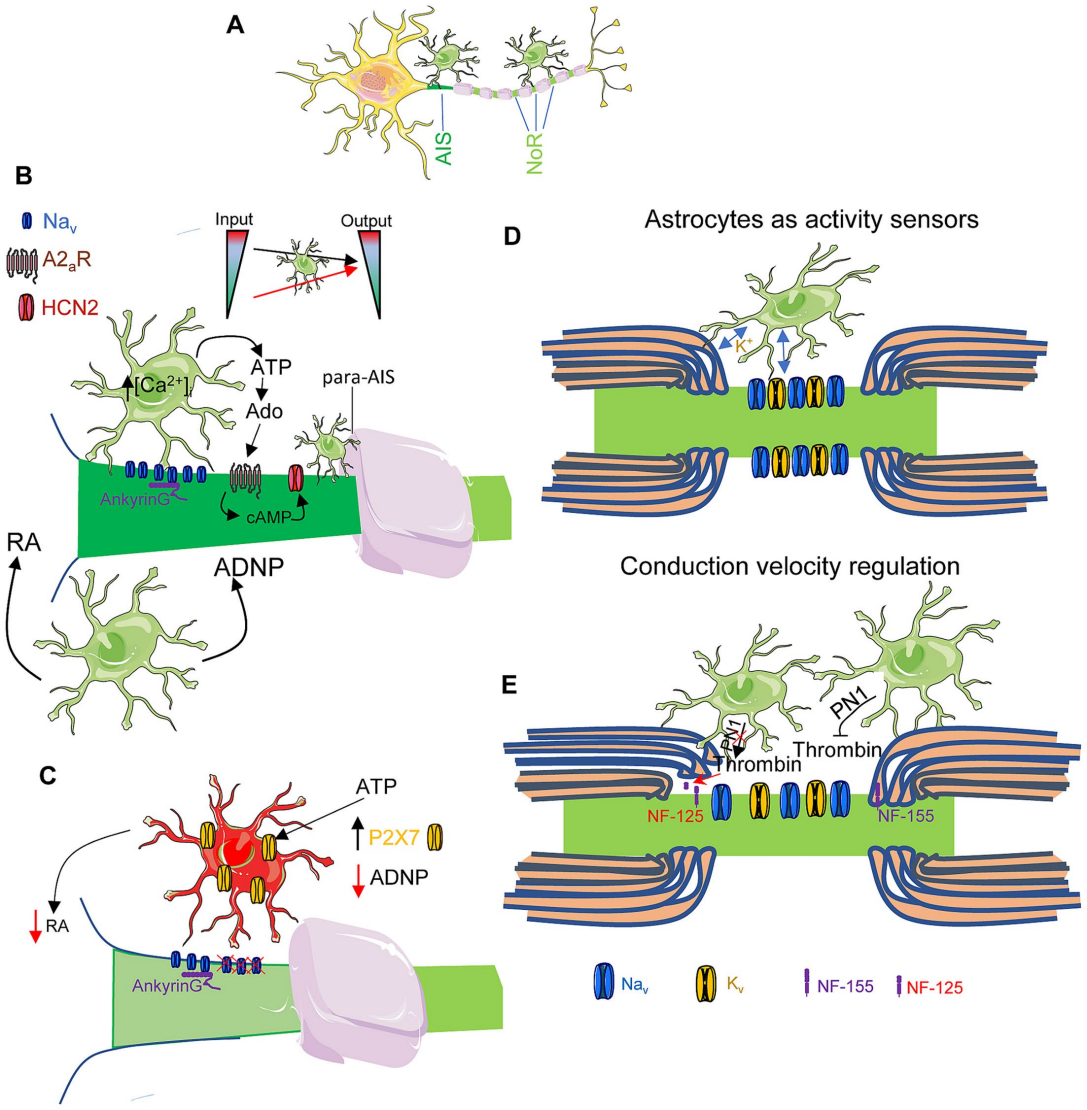
Axon initial segment and nodes of Ranvier regulation by astrocytes. **(A)** Schematic representation of astrocytes interaction with the AIS and nodes of Ranvier. **(B)** Physiological regulation of the AIS by astrocytes. Neuronal input modulates astrocytes, which modulate neuronal output through adenosine receptors and HCN2 channels at the AIS. Astrocytes can also interact with myelinating cells at the para-AIS, regulating their interaction and myelin sheath. Astrocytes also modulate AIS by retinoic acid (RA) secretion and activity-dependent neuroprotective protein (ADNP). **(C)** Pathological changes in astrocytes, such as P2X7 receptor increase or lower ADNP and RA secretion affect AIS composition and length. **(D,E)** Schematic representation of astrocytes interaction with the nodes of Ranvier. Astrocytes interact with nodes of Ranvier in several ways: (a) acting as activity sensors **(D)**, interacting with voltage-gated ion channels at the node and with myelinating cells and modulating homeostatic ion concentrations, and (b) modulating conduction velocity **(E)** through the regulation of the interaction between axon and myelinating cells mediated by neurofascin-155. Astrocytes secrete PN1 that inhibits thrombin and neurofascin-155 cleavage. Lack of PN1 secretion activates thrombin and cleaves neurofascin-155 to neurofascin-125, leading to increased nodal gap.

Although astrocyte interactions with axonal functional domains have been less extensively studied than their role in the tripartite synapse, emerging evidence highlights their involvement in modulating the axon initial segment (AIS) and nodes of Ranvier (Figure 1). Astrocytes influence AIS stability, structural organization, and plasticity through contact-mediated mechanisms, secreted factors, and the regulation of ion and neurotransmitter homeostasis. The existence of AIS-astrocyte contacts was described in retinal ganglion cells, where the distal portion of the initial segment is contacted by multiple astrocytic processes (Stone et al., 1995). Astrocytes secrete chondroitin sulfate proteoglycans (i.e., brevican, tenascin-R, neurocan) that interact with neurofascin-186 at the AIS to form a specialized extracellular matrix (Frischknecht et al., 2014). Although its precise function remains unclear, maturation of this extracellular matrix may contribute to stabilizing AIS plasticity after a critical period during which AIS length and position are dynamic in the visual cortex (Gutzmann et al., 2014). This matrix may also restrict lateral membrane diffusion at the AIS to preserve ion channels clustering or directly regulate the function of voltage-gated ion channels (Srinivasan et al., 1998; Evers et al., 2002). Astrocytes contacting axons near AIS and nodes of Ranvier additionally modulate neuronal excitability and conduction speed through astrocytes Ca^2+^-dependent ATP release (Lezmy et al., 2021). Neuronal input regulates astrocytic intracellular Ca^2+^, triggering ATP secretion that is subsequently converted to adenosine by ecto-nucleotidases. Adenosine then acts on A2a purinergic receptors at the AIS and nodes of Ranvier, allowing the fine-tuned control of neuronal excitability under varying levels of input (Figure 1B). Similarly, activation of Ca^2+^-permeable P2X7 purinergic receptors by ATP in hippocampal neurons reduces ankyrinG and Na_v_ channels density at the AIS through Ca^2+^ activation of calpain, an effect reversible by P2X7 receptor antagonists (Del Puerto et al., 2015). Age-related para-AIS remodeling has also been described in the first paranode distal to the AIS, with glia-dependent plasticity occurring between postnatal day 18 and 3 months of age in mice (Furusho and Terasaki, 2024), potentially affecting neuronal excitability. Astrocyte-derived soluble factors further contribute to AIS plasticity. Retinoic acid, necessary for neuronal physiology and synthesized by astrocytes, modulates AIS structure and protein density (Figure 1B). Reduced retinoic acid synthesis by astrocytes correlates with AIS shortening and decreased ankyrin-G levels, effects reversible by inhibiting the retinoic acid–degrading enzyme CYP26 (Benitez et al., 2024). Additionally, astrocyte-secreted ADNP (activity-dependent neurotrophic factor) is necessary to maintain AIS structure (Figure 1B) and decreased expression of ADNP in astrocytes (Figure 1C) leads to AIS shortening and reduced ankyrinG expression in hippocampal neurons (Benitez et al., 2024). ADNP fragments are secreted into the extracellular space (Furman et al., 2004) and internalized via dynamin-associated endocytosis (Ivashko-Pachima and Gozes, 2020). Treatment with the ADNP mimetic peptide, NAP, recovers AIS length and protein density (Benitez et al., 2024). ADNP also regulates the expression of multiple genes during embryogenesis (Mandel et al., 2007) and neuronal differentiation and maintenance (Mandel et al., 2008), stabilizes β-catenin during neural induction (Sun et al., 2020), interacts with end-binding proteins (EB1, EB3) to associate with microtubules (Ivashko-Pachima and Gozes, 2021), and contains an actin-binding domain that may allow direct or indirect interactions with actin-binding proteins (Ivashko-Pachima et al., 2022).

Astrocyte contacts at nodes of Ranvier were first described by Peters (1966) and were initially proposed to regulate ion homeostasis and myelin turnover (Hildebrand et al., 1993). More recent studies confirm that perinodal astrocytes serve as activity sensors and regulate both myelin structural dynamics and conduction velocity (Figures 1D,E). Astrocytes contacting nodes of Ranvier can also reversibly regulate myelin thickness and nodal gap length to optimize conduction speed. Impaired PN1 (thrombin inhibitor protease nexin1) release from astrocytes leads to paranodal loop detachment, nodal widening, and altered conduction. PN1 prevents neurofascin 155 cleavage and maintains the link between paranodal loop glial cells and axon, thereby influencing the integrity of paranodal attachments (Figure 1E). Reduced secretion of these inhibitors disrupts the linkage between paranodal loops and the axon, increases nodal length, and promotes sodium channel dispersion (Dutta et al., 2018).

Collectively, astrocyte-derived signals at the AIS and nodes of Ranvier exert profound regulatory effects on action potential initiation and propagation. These mechanisms enable dynamic modulation of neuronal function under physiological conditions and during axonal pathology.

## Microglia-mediated regulation of the AIS and nodes of Ranvier

**Figure 2:**
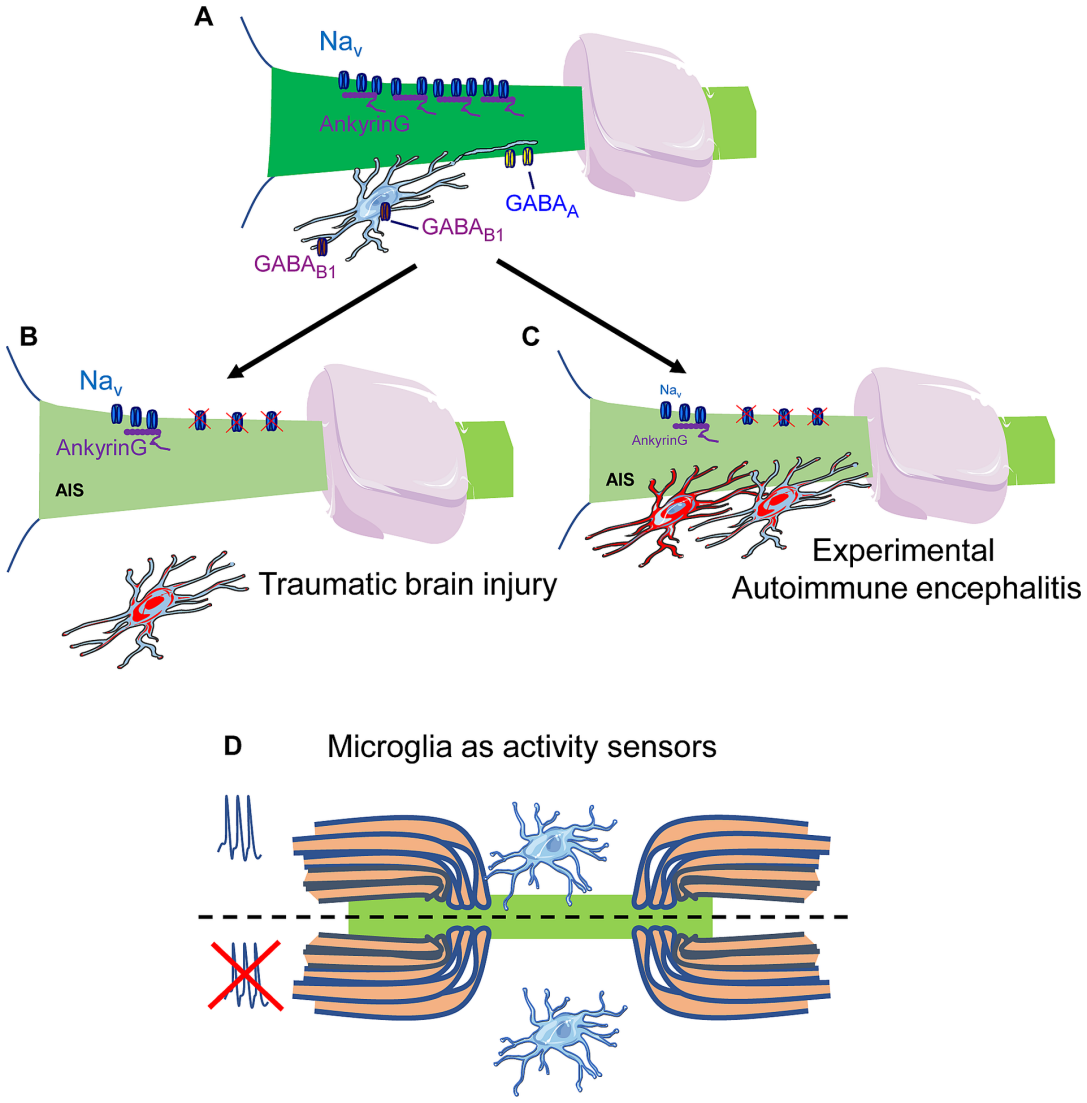
Regulation of the axon initial segment and nodes of Ranvier by microglia. **(A)** Schematic representation of microglia interaction with AIS. Microglia contact the AIS near GABA_A_ receptors. This interaction depends on an intact AIS and ankyrinG expression and the GABA_B1_ receptor in microglial cells in chandelier cell axo-axonic synaptogenesis. **(B,C)** Microglia-AIS interaction is disrupted after Traumatic brain injury **(B)**, while increased in experimental autoimmune encephalitis models **(C)**. Microglia act as activity sensors in nodes of Ranvier. **(D)** Microglia interaction with nodes of Ranvier depends on neuronal activity, which absence leads to microglia detachment through unknown mechanisms.

Microglia activation has long been associated with responses to brain or axonal damage, where microglia protect neural tissue from excitotoxicity and inflammation through cytokine release and phagocytic removal of debris. Microglia exert both neuroprotective functions, by remodeling synaptic connections and producing growth factors, and detrimental effects under inflammatory conditions (Hume, 2025). Recent studies have demonstrated that microglial cells also establish contacts with the AIS and nodes of Ranvier under physiological conditions (Baalman et al., 2015; Ronzano et al., 2021). Microglial cells were found in association with the AIS in cortical neurons (Figure 2A), preferentially non-GABAergic neurons, with approximately 8% of microglia contacting the AIS in the adult brain (Baalman et al., 2015). These contacts occur preferentially in excitatory neurons close to GABA_A_ receptor clusters in the AIS (Gallo et al., 2022), require an intact AIS structure, and are lost following mild brain injury, suggesting a physiological role for microglia in AIS modulation, either through regulation of AIS-associated synapses or by influencing local ion concentrations. Microglia also participate in the synaptogenic regulation of inhibitory circuits during postnatal brain development. Specifically, microglia regulate axo-axonic synapses formed by chandelier cells onto neocortical pyramidal neurons, a process dependent on microglial GABA_B1_ receptors and disrupted under neuroinflammatory conditions (Gallo et al., 2022). In human neurons carrying the epilepsy associated Nav1.2 mutation (Nav1.2-L1342P), co-culture with microglia results in decreased sodium channel expression, indicating a potential microglia-mediated suppression of hyperexcitability in mutant neurons (Que et al., 2023). Microglia also contact nodes of Ranvier in both rodents and humans (Zhang et al., 2019; Ronzano et al., 2021). Approximately 25% of nodes in the mouse spinal cord maintain stable microglial contacts, and this interaction is dynamically regulated by neuronal activity (Figure 2D), consistent with a role for microglia as sensors of neural activity (Ronzano et al., 2021). The mechanism appears more dependent on potassium conductances than sodium conductances. Blocking K^+^ channels with tetraethylammonium (TEA) reduces microglia–node contacts by ~40%, whereas sodium channel blockade with tetrodotoxin (TTX) produces only a ~ 20% reduction. Moreover, potassium regulation in the perinodal region depends on K^+^ channels both at the node and in microglia, which express THIK1 channels, and whose inhibition disrupts microglia–node interactions. Although inhibition of the microglial purinergic receptor P2Y12 does not affect baseline microglia–axon contacts, P2Y12 plays a protective role by promoting microglial wrapping of nodes in response to axonal injury, thereby contributing to axonal protection (Wu et al., 2024).

Our current understanding of how astrocytes and microglia modulate axonal function, from action potential initiation to propagation and conduction velocity, remains limited. Nonetheless, existing evidence is sufficient to establish that glial contacts at the AIS and nodes of Ranvier are as functionally significant as glial involvement at tripartite synapses. Elucidating the molecular and cellular mechanisms underlying glial regulation of axonal physiology between AIS and synaptic terminals is crucial for advancing our understanding of demyelinating and neurodegenerative diseases, as well as other mental and neurodevelopmental disorders.

## Brain diseases, glial cells, axon initial segment, and nodes of Ranvier

Growing knowledge of the composition, structure, and mechanisms underlying glial interactions with the axon initial segment (AIS) and nodes of Ranvier has expanded our understanding of their potential contributions to brain disorders associated with altered neuronal excitability. The role of astrocytes-AIS interactions remains relatively unexplored, and their involvement in disease is still poorly defined. Astrocytes become activated in response to various forms of brain damage or pathological conditions; however, altered neuronal activity itself can also trigger astrocytic activation. Increased astrocytic intracellular Ca^2+^, ATP release, and the subsequent activation of adenosine A2a receptors at the AIS enhance action potential firing frequency (Lezmy et al., 2021). However, under high input conditions, this frequency is reduced, suggesting that chronically elevated adenosine levels may disrupt action potentials in pathological states (Lezmy et al., 2021). The Alzheimer’s disease mouse model APP/PS1, characterized by the expression of human APP^695swe^ and presenilin 1 (PS1-ΔE9) mutants, presents shorter AIS and reduced ankyrinG levels in early postnatal development due to astrocytes transcriptomic alterations (Benitez et al., 2024). These changes include, among others, decreased Aldh1b1 and Rdh1 expression, two enzymes involved in retinoic acid synthesis, and reduced ADNP expression levels, both of which influence AIS physiology in wild-type neurons exposed to APP/PS1 astrocytes. The mechanism involves the P2X7 purinergic receptor, highly expressed in astrocytes and microglia, whose inhibition partially restores AIS properties both “*in vivo*” and “*in vitro*” (Figure 1C). Purinergic receptors are known to play key roles in glia–neuron communication in health and disease (Del Puerto et al., 2013), yet the detailed mechanisms are not fully understood. During aging, loss of progranulin (PGRN) and TMEM106B, a gene encoding a lysosomal membrane protein, results in increased glial activation and excessive lysosomal accumulation within the AIS of motor neurons (Feng et al., 2020), contributing to the development of frontotemporal lobar degeneration (FTLD). Additional age-associated changes in AIS and para-AIS organization have been described in mice (Benitez et al., 2024; Furusho and Terasaki, 2024). Further research is necessary to define the molecular mechanisms governing astrocyte-AIS interactions and to understand how their dysregulation contributes to neuropathological processes. Similarly, our understanding of astrocytes’ role in regulating nodes of Ranvier under pathological conditions remains limited. As noted above, astrocyte-nodes interactions are mediated through extracellular matrix molecules as well as gap junctions that link astrocytes and oligodendrocytes. Reduced mRNA levels of several extracellular adhesion molecules have been reported in the prefrontal cortex of patients with mood disorders and rodents (Miguel-Hidalgo, 2023). Altered astrocyte–oligodendrocyte communication via connexins has been implicated in multiple sclerosis, neuromyelitis optica, and Baló’s disease (Masaki, 2015). In addition, mutations in connexin 32 contribute to peripheral demyelination and neuropathy in Charcot–Marie-Tooth (Masaki, 2015). Such mutations or alterations in connexin-mediated signaling may impair oligodendrocyte trophic support by astrocytes, ultimately affecting myelination and the structural integrity of nodes of Ranvier. Conversely, the structure of nodes of Ranvier also influences astrocytic biology. Loss of ankyrinG has been shown to increase GFAP expression and astrocyte number (Jukkola et al., 2013). These astrocytic changes can, in turn, affect nodal function through altered release of gliotransmitters such as glutamate and ATP.

The involvement of microglia-AIS interactions has been studied in traumatic brain injury (TBI) and multiple sclerosis; however, the underlying molecular mechanisms remain poorly understood. In late experimental autoimmune encephalitis (EAE), AIS loss is accompanied by an increase in microglia-AIS contacts (Figure 2C) (Clark et al., 2016), in contrast to observations in TBI models (Figure 2B), where such contacts are reduced (Baalman et al., 2015). During both early and late stages of EAE, microglia exhibit a reactive morphology, and anti-inflammatory treatment attenuates AIS disruption (Clark et al., 2016). In a model of axonal crush-injury in motorneurons, the number of microglia contacting the AIS increases significantly (Tamada et al., 2021), concomitant with an increased number of mitochondria at the AIS, suggesting a potential mechanism supporting axonal survival and regeneration through both extracellular and intracellular modulation of AIS structure and function. These findings suggest that microglia can exert both deleterious and protective actions depending on the specific pathophysiological context.

Paranodal axo-glial interactions are disrupted in Parkinson’s disease tissue, showing diffuse microglial inflammation in the absence of demyelination, as well as in multiple sclerosis brains before demyelination, indicating that microglial inflammation may precede alterations in the nodes of Ranvier (Howell et al., 2010). Similar observations have been made in the EAE multiple sclerosis model, where microglia activation precedes the onset of clinical symptoms (Howell et al., 2010). Expression of Na_v_1.6 voltage-gated sodium channels is disrupted in 20–30% of nodes, an effect that is reversible upon attenuation of inflammation. Additional studies have linked this microglia-associated inflammatory state to the loss of the P2Y12 purinergic receptor expression in this microglia, resulting in loss of microglia homeostatic phenotype (Zrzavy et al., 2017). Loss of this homeostatic phenotype is similarly observed across other neurodegenerative conditions of the CNS (Butovsky et al., 2014).

In conclusion, glial cells play roles far beyond structural and metabolic support for neurons. Emerging evidence demonstrates that astrocytes and microglia actively interact with axonal excitable domains, AIS and nodes of Ranvier, extending their influence beyond the classical tripartite synapse and contributing to the modulation of neuronal function in both physiological and pathological contexts. Further research is needed to elucidate the molecular mechanisms governing glial regulation of axonal excitable domains, their role in neuronal excitability and axonal domain plasticity, and the bidirectional regulation between neuronal activity and glial function.
